# Extract of *Allium tuberosum Rottler ex Spreng* Promoted the Hair Growth through Regulating the Expression of IGF-1

**DOI:** 10.1155/2015/413538

**Published:** 2015-05-20

**Authors:** Ki Moon Park, Dong Woo Kim, Seung Ho Lee

**Affiliations:** ^1^Department of Food Science and Biotechnology, Sungkyunkwan University, Suwon 440-746, Republic of Korea; ^2^Major of Nano-Bioengineering, Incheon National University, Incheon 406-772, Republic of Korea

## Abstract

*Allium tuberosum Rottler ex Spreng* (ATRES) has been used as a traditional medicine for the treatment of abdominal pain, diarrhea, and asthma. In this study, we investigated the hair growth promoting activities of ATRES on telogenic C57BL6/N mice. Hair growth was significantly increased in the dorsal skin of ethanol extract of ATRES treated mouse group compared with the control mouse group. To enrich the hair promoting activity, an ethanol-insoluble fraction was further extracted in sequence with *n*-hexane, dichloromethane, ethyl acetate, *n*-butanol, and distilled water. Interestingly, we found that extraction with *n*-butanol is most efficient in producing the hair promoting activity. In addition, the soluble fraction of the *n*-butanol extract was further separated by silica gel chromatography and thin layer chromatography (TLC) resulting in isolating four single fractions which have hair growth regeneration potential. Furthermore, administration of ATRES extracts to dorsal skin area increased the number of hair follicles compared with control mouse group. Interestingly, administration of ATRES extract stimulated the expression of insulin-like growth factor-1 (IGF-1) but not of keratin growth factor (KGF) or vascular endothelial growth factor (VEGF). Taken together, these results suggest that ATRES possesses strong hair growth promoting potential which controls the expression of IGF-1.

## 1. Introduction

Hair plays an important role in body functions by protecting the head and maintaining the temperature of the head. Because of the increasing importance of hair in the field of beauty care, many researchers have focused on developing therapeutics for enhancing hair growth or preventing depilation [1]. A unique feature of hair growth is its cyclicity, which represents a growth phase (anagen), an involution phase (catagen), and a resting phase (telogen) [2]. For this reason, many agents that can induce anagen from telogenic hair are being screened for people suffering from hair loss. Currently, only two FDA approved drugs, finasteride and minoxidil, exist for hair loss patients [3]. However, finasteride and minoxidil have limitations in their therapeutic use due to side effects [4]. Consequently, a significant amount of effort has been focused on developing more desirable agents that can prevent hair loss.

Since the demand for novel therapeutics that provide safe hair growth enhancing results is growing, although most reports used the total extract of a herb to show the hair growth promoting activity without separating the substance into a single compound, many traditional herbs have been screened to determine whether or not they promote hair growth activity. It has been reported that* Fructus panax* ginseng extract has shown hair regeneration activity in C57BL6/N mice [5] and* Eclipta alba* extract was also reported to have hair growth promoting activity [6]. It was revealed that* Polygonum multiflorum* extract induces hair growth in resting hair follicles by upregulating Shh and beta catenin expression [7].

ATRES is an* Allium* species known as Chinese chive that is widely distributed in East Asia. ATRES have been used for treating abdominal pain, diarrhea, hematemesis, and asthma in oriental medicine [8]. ATRES was also reported to have choline acetyltransferase activity which is responsible for the synthesis of the neurotransmitter acetylcholine [9].* Allium tuberosum* seeds extract was reported to have aphrodisiac properties [10] and, interestingly, one report showed hair promoting activity by directly administrating crude onion juice (*Allium cepa* L.) to alopecia areata patients [11]. However, there are no reports about the effect of ATRES on hair growth and mechanisms of ATRES on hair growth remain unexplored. In the present report, we investigated the hair growth promoting activity of extracts of ATRES and its mechanism of action. In addition, we optimized the extraction method for isolating the fraction that contains the highest hair growth promoting activity from ATRES.

## 2. Material and Methods

### 2.1. Reagents

ATRES was obtained from a local Nonghyup market in Gangwon-do, Korea. Minoxidil was obtained from Hyundai Pharm. Co. Ltd. (Cheonan, Korea) and 1-(4,5-dimethylthiazol-2-yl)-3,5-diphenyltetrazolium bromide (MTT) was obtained from Sigma-Aldrich (Yongin, Korea).

### 2.2. Cell Culture and Cytotoxicity Assay

Human hair dermal papilla cells (HHDPC) and human keratinocyte HaCaT cells were obtained from ScienCell Research Laboratories (Carlsbad, CA, USA) and American Type Culture Collection (ATCC, Manassas, VA, USA), respectively. The cells were maintained in Dulbecco's Modified Eagle's Medium (DMEM) (WelGENE Inc., LM 001-05) supplemented with 10% fetal bovine serum (FBS) (HyClone, SH30919.03), penicillin (100 U/mL), and streptomycin (100 ug/mL). Next, 1.5 × 10^5^ cells/well were seeded in a 96-well plate and cultured for 24 h. The extracts were added to culture media and incubated at 37°C in CO_2_ incubator for 24 h, 48 h, and 72 h. MTT solution was then added and incubated for 2 h. Dimethyl sulfoxide (DMSO, Sigma-Aldrich, Yongin, Korea) was used to dissolve the formazan crystals. The absorbance was measured at 540 nm with a microplate reader (Bio-Rad, Hercules, CA, USA).

### 2.3. Extraction and Fractionation

About 300 g of ATRES was extracted with 2000 mL ethanol at 40°C for 48 h. The extract was concentrated using a vacuum evaporator. It was then dissolved with 40% ethanol for subsequent treatment in the* in vivo* experiments. The yield of ethanol extraction of ATRES was 19.5% (w/w, dry weight 58.5 g). The insoluble fraction from the ethanol extract was then used for extraction by* n*-hexane and the insoluble fraction of the* n*-hexane extraction was used for the next extraction with dichloromethane. The insoluble fraction of dichloromethane extraction was used for the extraction with ethyl acetate and the insoluble fraction of ethyl acetate was then used for the extraction with* n*-butanol. Finally, the insoluble fraction of* n*-butanol was extracted with distilled water (DW). Each soluble fraction was concentrated, dried, and used for investigating the hair growth promoting activity. The yield of each dried extract was 3.27 g, 0.348 g, 0.21 g, 16.14 g, and 41.04 g, respectively.

### 2.4. Silica Gel Chromatography

About 700 g of C18 silica gel was packed into an open column (50 mm diameter and 600 mm length, Bio-Rad Laboratories, Berkeley, CA, USA). Three hundred milliliters of methyl chloride/methanol (5 : 1) buffer was preloaded into the column and then the* n*-butanol soluble fraction was applied to the silica column and eluted with 400 mL of the methyl chloride/methanol (5 : 1) buffer. Each of the five fractions was tested using thin layer chromatography (TLC).

### 2.5. Thin Layer Chromatography

Each of the five fractions from the silica gel chromatography was confirmed by thin layer chromatography (TLC). The TLC plate (TLC silica gel 60, Merk Co.) was developed in chloroform/methanol/water (3 : 3 : 1) and it was visualized by heating at 80°C for 30 min after dipping in ethyl alcohol/sulfuric acid/anisaldehyde/acetic acid (90 : 5 : 1 : 1). After confirming the component, the fractionated samples, which contained the same component, were mixed, dried, and used for testing the hair growth promoting activity.

### 2.6. *In Vivo* Analysis of the Hair Growth Promoting Activity of the ATRES Extract

Six-week-old male C57BL6/N mice were obtained from Orient Bio (Eumsung, Korea) and housed in stainless steel cages under controlled temperature (23 ± 3°C), humidity (55 ± 10%), and photoperiod (12 h cycles of light and dark). All the animals were fed standard mice chow (Orient Bio) and water,* ad libitum*. For acclimation, all the mice were kept for one week before starting the experiment. These experiments were conducted in accordance with the Animal Research Committee of Sungkyunkwan University. The mice (*n* = 6 per group) were anesthetized with an intraperitoneal injection of a mixture of Rompun (Bayer, Leverkusen, Germany) and Zoletil (Virabc, Carros, France). All the mice were shaved using animal clippers and further hair was eliminated using hair removal cream (Oxy Reckitt Benckiser, Seoul, Korea). To check the hair growth promoting activity, an ethanol extract of ATRES (3% or 5% in 150 uL of 40% ethanol) was applied topically to the dorsal back area every day for 14 days. The same volume of 40% ethanol and minoxidil (MXD; 3% or 5%) was used for the negative and positive controls, respectively. Soluble extracts (3% in 150 uL of 40% ethanol) of other solvents (*n*-hexane, dichloromethane, ethyl acetate,* n*-butanol, and DW) were used for* in vivo* analysis.

### 2.7. Expression of Growth Hormones in the Dorsal Skin Area and HaCaT Cell

The dorsal skin was isolated at the end of the animal study. HaCaT cell was treated with ethanol extract and incubated at 37°C in CO_2_ incubator for 24 h. Total RNA was isolated with TRIzol reagent (Invitrogen, Carlsbad, CA) according to the manufacturer's protocol. Changes in the expression levels of IGF-1, VEGF, and KGF were calculated using a real-time PCR machine (2640A; Takara) as previously described [12]. The mRNA levels were normalized to beta actin. Specific oligonucleotide primers are described in Table 1.

### 2.8. Histochemistry

The dorsal skin area (13 cm^2^) was excised between the fore leg and the hind leg after being treated with ATRES extract. Each dorsal skin area was then fixed in 4% paraformaldehyde (Sigma-Aldrich, St. Louis, MO, USA) and embedded in paraffin blocks. Next, 4 *μ*m sections were stained with hematoxylin and eosin (H&E) and the representative area was photographed via light microscopy (Olympus CX 31; Olympus, Tokyo, Japan).

## 3. Results

### 3.1. The Effect of the Ethanol Extract of ATRES on Hair Regeneration

**Figure 1:**
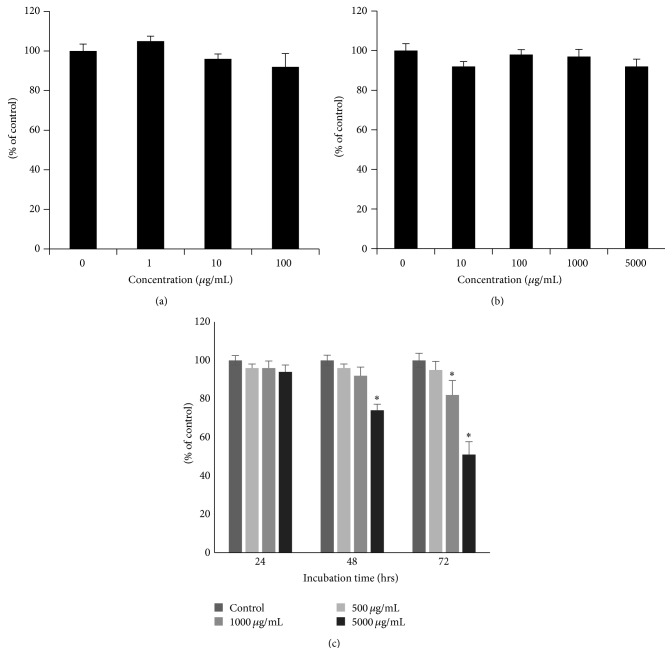
Cytotoxicity of the ethanol extract of ATRES. Ethanol extracts of ATRES were added to human dermal papilla cells (0–100 ug/mL) and human keratinocyte HaCaT cells (0–5000 ug/mL). The cell viability of the human dermal papilla cells (a) and the human keratinocyte HaCaT cells (b) were measured using an MTT assay for 24 h incubation. The cell viability of HaCaT cells were further determined by adding the ATRES during 72 h incubation (c). Each data value is indicated as the means ± SE for three independent experiments. *∗* indicates *P* < 0.05 as compared to the control.

**Figure 2:**
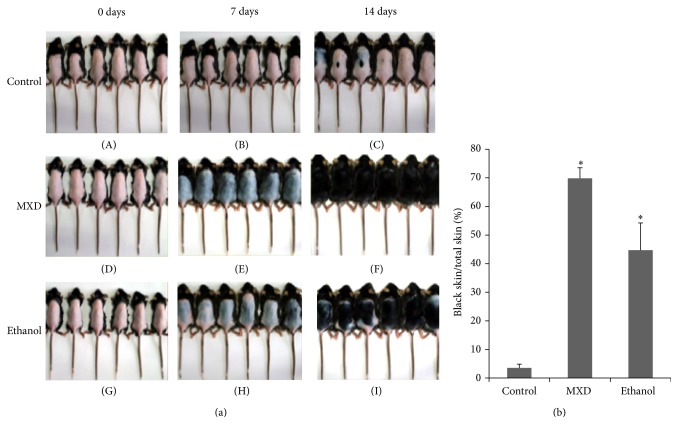
Ethanol extract of ATRES enhanced hair growth. (a) Telogenic mice (*n* = 6) treated with ethanol extract of ATRES (5% in 150 uL of 40% ethanol) ((G), (H), and (I)) and 5% of minoxidil ((D), (E), and (F)) were compared with control (150 uL of 40% ethanol treated) ((A), (B), and (C)) groups over a 2-week period. (b) Hair growth was significantly promoted by administration of the ethanol extract of ATRES when compared to the control mouse groups (*P* < 0.001). Differences were calculated by measuring the size of the complete black area (black skin) using the Image J program. The terms MXD and ethanol refer to the minoxidil extract treated mouse group and the ethanol extract treated mice group, respectively. Each data value is indicated as the mean ± SE. *∗* indicates *P* < 0.001 as compared to the control group.

**Figure 3:**
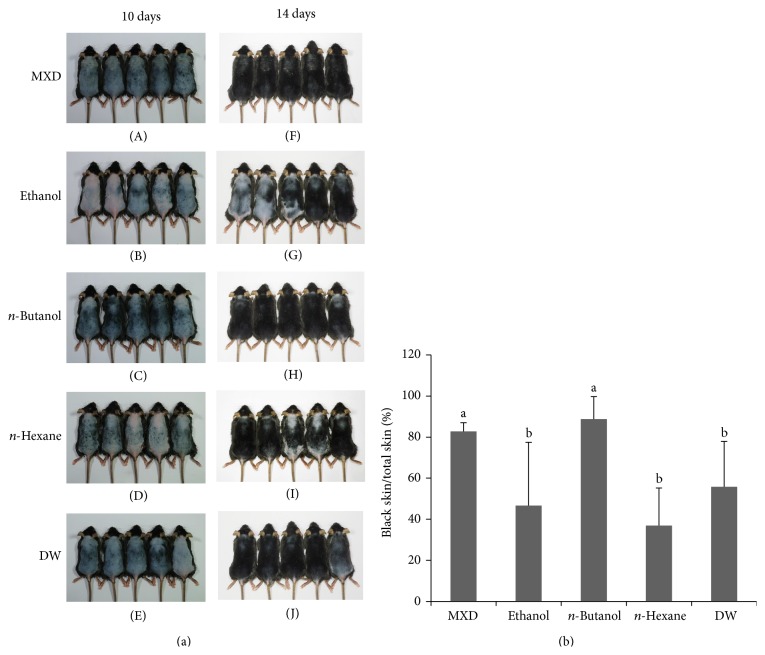
The* n*-butanol extract of ATRES showed the highest level of hair growth promoting activity. The insoluble fraction of the ethanol extract was first used for the subsequent extraction with different solvents. (a) The hair growth promoting activity was photographed at 10 days ((A), (B), (C), (D), and (E)) and 14 days ((F), (G), (H), (I), and (J)) after treatment and compared with the mouse groups that were administrated ethanol ((B), (G)),* n*-butanol ((C), (H)),* n*-hexane ((D), (I)), and DW ((E), (J)) extracts (3% in 150 uL volume of 40% ethanol). (b) Anagenic areas (complete black area) at 14 days of treatment were calculated using the Image J program and the* n*-butanol extract treated group showed the most enhanced hair growth. Data are presented as the means ± SD (*n* = 5) and different letters of (b) indicate significant differences (*P* < 0.05) between the groups.

Ethanol extract of ATRES was first administrated to human dermal papilla cells and human keratinocyte HaCaT cells to check for cytotoxicity* in vitro*. The number of human dermal papilla cells was not affected by adding up to 100 ug/mL of the ethanol extract of ATRES (Figure 1(a)); moreover, no significant change was seen in the growth of human keratinocyte HaCaT cells by adding up to 500 ug/mL of the ethanol extract of ATRES (Figure 1(c)). After confirming the cytotoxicity of the ethanol extract of ATRES, the ethanol extract of ATRES was topically administrated to the trimmed dorsal skin area to investigate the hair growth promoting potential of ATRES for 2 weeks. Since the C57BL6/N strain of mice has time-synchronized hair growth cycles, the hair growth promoting activity of the ethanol extract of ATRES was investigated by comparing the area that changes color from pink (telogen phage) to gray (anagen phage) after the trimmed dorsal skin area was treated with the ethanol extract of ATRES. As shown in Figure 2, the trimmed dorsal skin area in the control group remained in the telogen phase (pink) the longest (until day 14); however, both the minoxidil (MXD) (5%, w/v) treated group and the ethanol extract of ATRES (5%, w/v) treated group exhibited light gray skin 10 days after depilation and many hair shafts were detected within 14 days. During the anagen phase, the trimmed dorsal skin area (complete black area), which was measured using the Image J processing program, was compared 14 days after treatment and the group that was treated with the ethanol extract of ATRES showed a significantly enlarged anagenic area in comparison to the control group (Figure 3(b)). These results indicate that ATRES has strong hair growth promoting activity.

### 3.2. Hair Growth Promoting Effect of the Fraction Extracts of ATRES

After confirming the hair growth promoting activity of the ethanol extract of ATRES, the insoluble fraction of the ethanol extract was used for further extraction with* n*-hexane, dichloromethane, ethyl acetate,* n*-butanol, and distilled water (DW), successively. Hair growth enhancing potential was tested by administering each of the soluble extracts to the telogenic mice. Although the soluble fraction of dichloromethane and ethyl acetate extraction showed no hair growth promoting activity* in vivo* (data not shown), the groups that were administrated* n*-butanol,* n*-hexane, and DW extracts showed enhanced hair growth 14 days after treatment (Figure 3). Interestingly, the groups treated with* n*-butanol extract showed the most enhanced hair growth promoting activity (Figure 3(b)) when compared to the treatments with the ethanol,* n*-hexane, and DW extracts. These results suggest that* n*-butanol extraction could be the most efficient way to isolate the ATRES components that have hair promoting activity.

### 3.3. Further Fractionation of the* n*-Butanol Soluble Fraction by Silica Gel Chromatography

**Figure 4:**
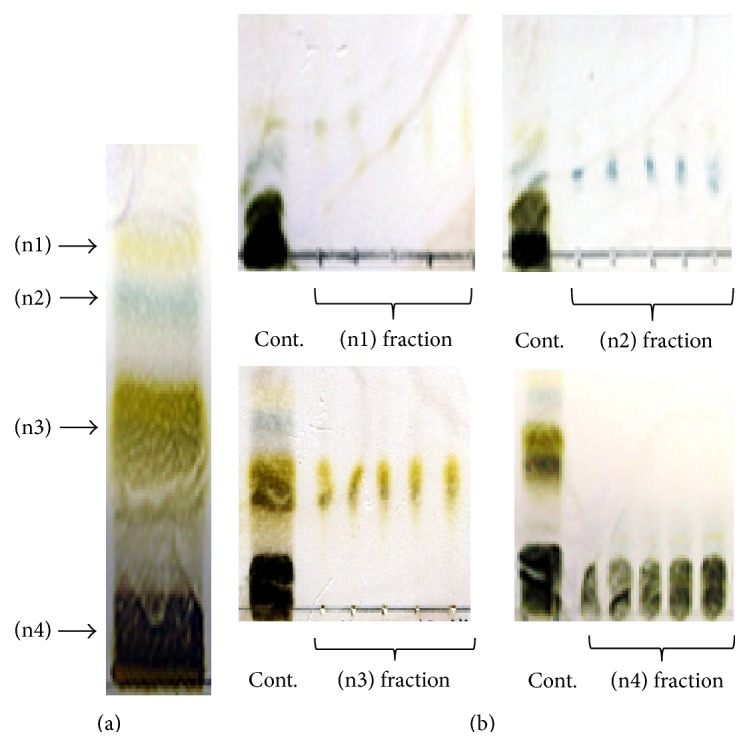
The* n*-butanol soluble fraction was further separated using silica gel chromatography. (a) The* n*-butanol extract was dipped onto the TLC plate and developed. To isolate the four different subfractions (n1, n2, n3, and n4), the* n*-butanol soluble fraction was loaded onto the silica gel column and eluted to 400 vials (1 mL per vial). (b) The representative TLC figure for each fraction (n1, n2, n3, and n4) is shown. Elutes in each of the every five vials were tested via TLC. Elutes that showed the same TLC result were mixed and then dried. Cont. refers to the column that was loaded with the* n*-butanol extract.

**Figure 5:**
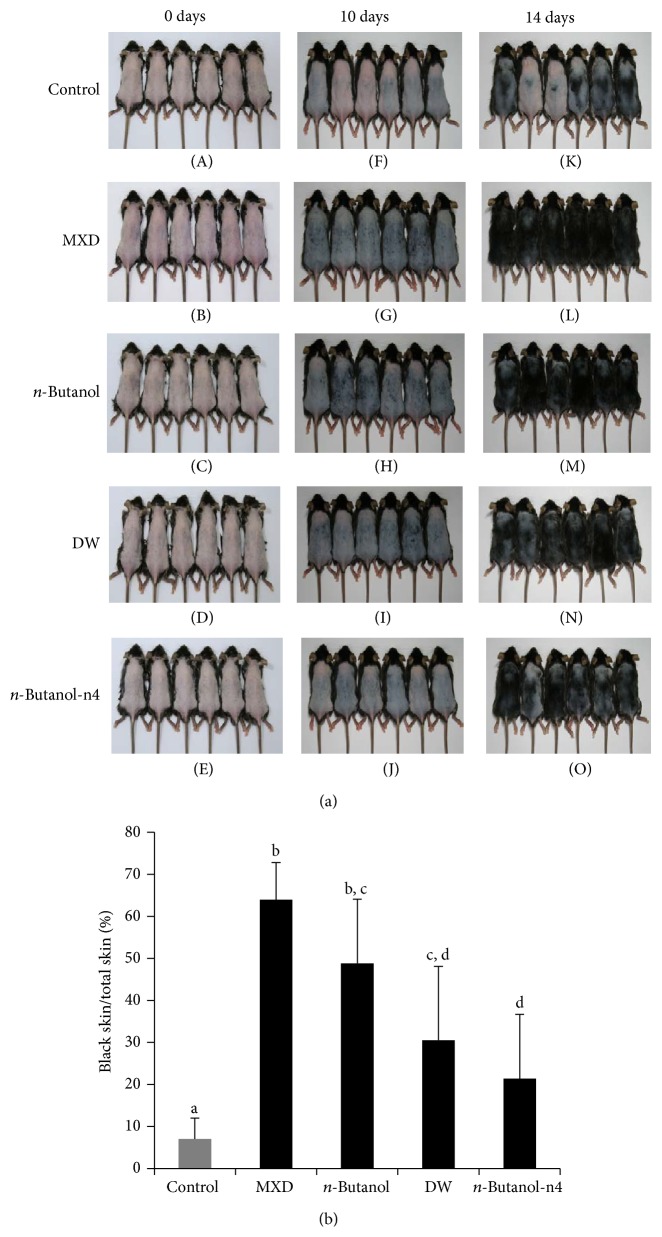
A comparison of the hair growth promoting potential of* n*-butanol-n4 fraction extract* in vivo*. (a) The* n*-butanol-n4 fraction (3%, w/v) ((E), (J), and (O)) was administrated to telogenic mice to compare its hair growth promoting potential with the* n*-butanol (3%, w/v) ((C), (H), and (M)) and DW (3%, w/v) ((D), (I), and (N)) extracts. MXD (3%, w/v) was used as the positive control ((B), (G), and (L)) and 40% ethanol was used as the negative control ((A), (F), and (K)). The dorsal skin area (*n* = 6) was photographed at 0 days, 10 days, and 14 days after treatment. (b) The anagenic areas (complete black area) 14 days after treatment were calculated by using the Image J processing program. The data are presented as the means ± SD (*n* = 6) and the different letters in (b) indicate significant differences (*P* < 0.05) between the groups.

Since the* n*-butanol soluble fraction showed the most enhanced hair promoting activity (Figure 3(b)), it was used for further fractionation. TLC analysis revealed that the* n*-butanol soluble fraction consisted of four different components: n1, n2, n3, and n4 (Figure 4(a)), and these four fractions were separated by silica gel chromatography (Figure 4(b)). Since the n4 fraction showed the highest hair growth promoting activity among the four different* n*-butanol fractions* in vivo* (data not shown), the hair growth promoting activity of* n*-butanol-n4 fraction was compared with the hair growth promoting activity of MXD,* n*-butanol, and the distilled water soluble fraction (Figure 5). As shown in Figure 5, although the hair growth promoting activity of the* n*-butanol-n4 fraction was lower than that of the* n*-butanol extract, the findings clearly show that the hair growth activity was significantly increased in the* n*-butanol-n4 fraction treated groups (Figure 5(b)) in comparison to the control groups (*P* < 0.05).

### 3.4. The Effect of ATRES Extract on IGF-1 Expression in C57BL6/N Mice

**Figure 6:**
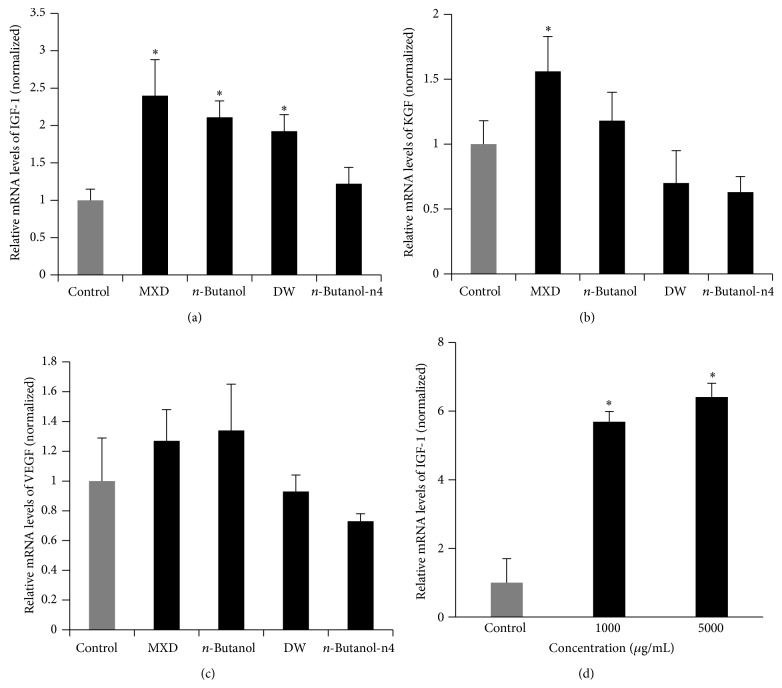
Administration of ATRES extract increased the mRNA expression of IGF-1 on the dorsal skin area. Total RNA was isolated from the dorsal skin area of the mice treated with 40% ethanol (negative control), MXD (3%, w/v, positive control), the* n*-butanol (3%, w/v) extract, the DW (3%, w/v) extract, and the* n*-butanol-n4 fraction (3%, w/v). Total RNA was isolated from HaCaT cells treated with 40% ethanol (negative control) and ethanol extract. The relative levels of expression of IGF-1 ((a), (d)), KGF (b), and VEGF (c), normalized to beta actin, were determined by qRT-PCR. The data were analyzed by ANOVA, and the Student-Newman-Keuls test was then conducted. The data are presented as the means ± SD (*n* = 3). *∗* indicates a statistically significant difference compared with the control group (*P* < 0.05).

To elucidate the underlying mechanism of the hair growth promoting events of the ATRES treatments, we compared the mRNA expression levels of several growth factors in the dorsal area treated with MXD,* n*-butanol, DW, and* n*-butanol-n4 fraction extracts by qRT-PCR (Figure 6). The mRNA expression levels of VEGF and KGF were not affected by administration of the ATRES extracts (Figures 6(b) and 6(c)). However, the dorsal skin area of the mice treated with the extract of* n*-butanol and distilled water showed increased levels of IGF-1 mRNA (*P* < 0.05) (Figure 6(a)). In addition, administration of extract of ethanol to HaCaT cell induced the levels of IGF-1 mRNA (*P* < 0.05) (Figure 6(d)).

### 3.5. The Effect of ATRES Extracts on the Number of Hair Follicles

**Figure 7:**
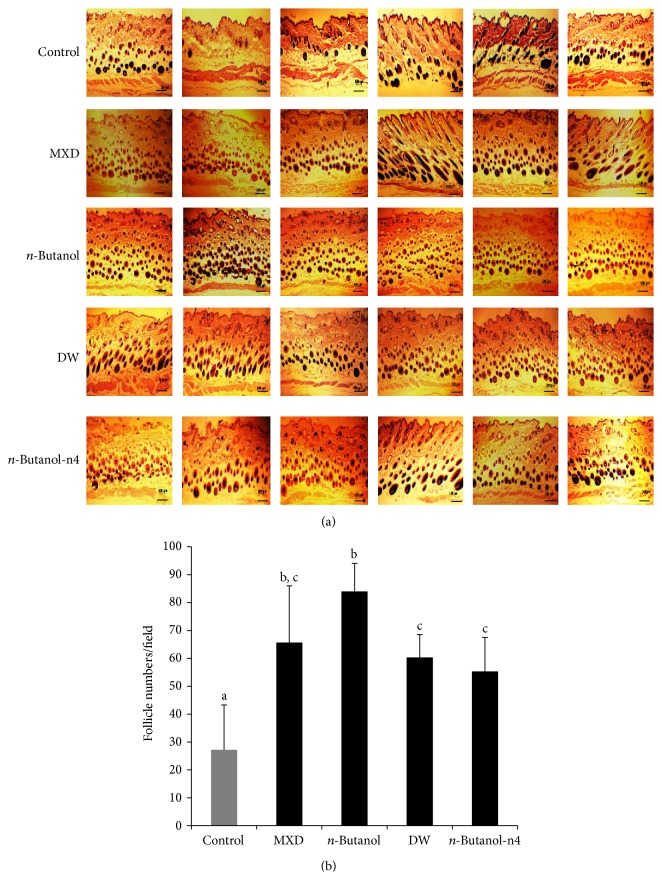
Histochemical analysis of hair follicles in the dorsal skin area treated with the ATRES extracts. After the dorsal skin area of telogenic mice was treated with 3% (w/v) of MXD,* n*-butanol extract, DW extract, and* n*-butanol-n4 fraction, the area was isolated and fixed with 4% paraformaldehyde. Each section was stained with hematoxylin and eosin (a) and then the number of hair follicles was counted (*n* = 6) (b). The data are presented as the means ± SD (*n* = 6) and different letters in Figure 7(b) indicate significant differences between the groups (*P* < 0.05). Scale bars: 200 *μ*m.

To investigate changes in the number of hair follicles after administration of several fractions of ATRES extract, we histologically compared the dorsal skin area by H&E staining (Figure 7(a)). Although the number of hair follicles in both the DW and* n*-butanol-n4 fraction treated groups was significantly increased in comparison to the control group (*P* < 0.05), the* n*-butanol extract treated group showed the greatest increase in the number of hair follicles (Figure 7(b)). These data indicate that the* n*-butanol extract was most effective in hair follicle enrichment.

## 4. Discussion

This study examined the hair growth promoting potential of ATRES through topical administration of extracts to the dorsal skin of C57BL6/N mice. ATRES is a well-known edible plant and it has been used in making traditional fermented food such as Kimchi in Korea. It was reported that consumption of* Allium* vegetables can reduce oxidative stress [13] and consumption of high levels of* Allium* vegetables reduced the risk for gastric cancer [14]. Although several studies have reported on the functional properties of ATRES [9, 15, 16], to our knowledge, this is the first report that demonstrates the hair growth promoting activity of ATRES. Administration of 5% of ethanol extracts of ATRES to the dorsal skin area of the C57BL6/N mice induced as much hair growth as found in the 5% minoxidil treated (MXD) group, while less visible hair growth was seen in the control groups. Since there is a growing need to develop novel therapeutics that show higher hair growth activity with lower side effects, it seems essential to determine the sufficient extraction methods by isolating the single compound that has therapeutic activity. After confirming the hair growth potential of the ethanol extract of ATRES, we focused on isolating the single compound that has hair growth promoting potential. Although the soluble fraction of the ethanol extracts showed hair growth promoting activity, we thought that the insoluble fraction of the ethanol extracts might contain components that show higher hair growth promoting activity than the soluble fractions. Since the solubility of a component usually depends on its polarity, the insoluble fractions of the ethanol extract were sequentially extracted with* n*-hexane, dichloromethane, ethyl acetate,* n*-butanol, and distilled water. Interestingly, the soluble fraction of the* n*-butanol extract proved to have the most hair growth promoting activity among those extracts* in vivo* (Figure 3). Recently, many research groups have suggested that traditional foods, plants, and grains, and so forth, have new pharmaceutical potential, but those investigators have mostly used soluble fractions extracted with one solvent and they discarded the insoluble fractions of the extract [7, 17]. That approach seems to be sufficient enough to show whether or not pharmaceutical activity exists in those materials; however, many additional steps should be considered in developing therapeutics using those materials. First of all, the most efficient extraction method should be determined and the identification of a single component is needed to enhance hair growth activity and reduce side effects. In our study, the* n*-butanol soluble fraction was further separated by silica gel chromatography; consequently, four different subfractions were isolated. Interestingly, each of the subfractions of the* n*-butanol extract showed hair growth enhancing activity* in vivo* (data not shown), but the hair growth promoting activity of each of the* n*-butanol extract subfractions was lower than that of the* n*-butanol extract (Figure 5). Although each of the four subfractions should be further identified by mass analysis or NMR, they all seem to have a cooperative effect in maximizing hair growth promoting activity.

Various growth factors have been proposed as controllers in hair growth. For example, VEGF, which is a well-known stimulator in angiogenesis, was reported to be a modulator in hair growth [18]. Ginsenoside-mediated hair growth also seems to be mediated by the upregulation of VEGF expression [19]. Some studies have suggested that keratinocyte growth factor (KGF) signaling is important in hair follicle development [20, 21]. It has also been reported that hair growth was increased when recombinant KGF was administrated to nude mice [22]. IGF-1 signaling was reported to be important in follicular proliferation and tissue remodeling [23, 24] as well as in inhibiting hair cell apoptosis [25]. Therefore, the expression of these growth factors has been checked when the hair growth promoting activity was detected by administration of therapeutic candidates. In our study, the expression of VEGF and KGF was not changed in the mouse groups treated with the* n*-butanol and DW extracts (Figure 6). Although the mRNA expression of IGF-1 was not changed in the mouse group treated with the n4 fraction of the* n*-butanol extract, the mRNA expression of IGF-1 was significantly increased in the mouse group that was administered the* n*-butanol and DW extracts, in comparison to the control mouse groups (Figure 6). Interestingly, administration of ethanol extract of ATRES to HaCaT cell significantly increased the expression of IGF-1 (Figure 6(d)). In addition, the number of hair follicles was also found to significantly increase in the mouse groups treated with the n4 fraction of the* n*-butanol extract and the* n*-butanol and DW extracts (Figure 7). These data suggest that the hair growth promoting activity of ATRES may be mediated by IGF-1 signaling. When these findings are taken together, our study strongly suggests that ATRES has hair growth promoting potential* in vivo* and that the* n*-butanol extraction with silica gel chromatography method could be helpful way in developing a drug from ATRES. Further study should focus on identification, and functional studies of the four subfractions that were separated from the* n*-Butanol extract by silica gel chromatography should be conducted.

## 5. Conclusion

The present study shows that ATRES has strong hair promoting activity. We determined that butanol extraction is the most efficient way to enrich the hair promoting activity. In addition, four single components, which have hair growth promoting activity, were isolated by silica gel chromatography. Administration of ATRES extract to dorsal skin area increased the number of hair follicles and expression of IGF-1. Our study is the first attempt to investigate the hair promoting potential and its mechanism of action of ATRES extract.

## Figures and Tables

**Table 1:** Nucleotide sequence of the primers.

Gene	Sequences
Human IGF-1	
Forward	5′-TTCTGGCCCCAGGATAACAC-3′
Reverse	5′-GTTTCCCAGGAGTGGTGGAA-3′
Mouse IGF-1	
Forward	5′-CACTGACATGCCCAAGACTCAGA-3′
Reverse	5′-TCCGAGTTGCCTCCGTTACC-3′
Mouse VEGF	
Forward	5′-CTGGATATGTTTGACTGCTGTGGA-3′
Reverse	5′-GTTTCTGGAAGTGAGCCAATGTG-3′
Mouse KGF	
Forward	5′-TGGTACCTGAGGATTGACAAACGA-3′
Reverse	5′-CCTTTGATTGCCACAATTCCAAC-3′
Beta actin	
Forward	5′-CATCCGTAAAGACCTCTATGCCAAC-3′
Reverse	5′-ATGGAGCCACCGATCCACA-3′

## References

[1] Stenn K. S. (1991). Induction of hair follicle growth. *Journal of Investigative Dermatology*.

[2] Greco V., Chen T., Rendl M. (2009). A two-step mechanism for stem cell activation during hair regeneration. *Cell Stem Cell*.

[3] D'Amico A. V., Roehrborn C. G. (2007). Effect of 1 mg/day finasteride on concentrations of serum prostate-specific antigen in men with androgenic alopecia: a randomised controlled trial. *The Lancet Oncology*.

[4] Shapiro J. (2013). Current treatment of alopecia areata. *Journal of Investigative Dermatology Symposium Proceedings*.

[5] Park S., Shin W.-S., Ho J. (2011). *Fructus panax* ginseng extract promotes hair regeneration in C57BL/6 mice. *Journal of Ethnopharmacology*.

[6] Datta K., Singh A. T., Mukherjee A., Bhat B., Ramesh B., Burman A. C. (2009). Eclipta alba extract with potential for hair growth promoting activity. *Journal of Ethnopharmacology*.

[7] Park H.-J., Zhang N., Park D. K. (2011). Topical application of *Polygonum multiflorum* extract induces hair growth of resting hair follicles through upregulating Shh and beta-catenin expression in C57BL/6 mice. *Journal of Ethnopharmacology*.

[8] Jiangsu New Medicinal College (1979). *The Dictionary of Chinese Herbal Medicines*.

[9] Kim M. J., Choi S. J., Kim H. K. (2007). Activation effects of *Allium tuberosum* Rottl. on choline acetyltransferase. *Bioscience, Biotechnology and Biochemistry*.

[10] Guohua H., Yanhua L., Rengang M., Dongzhi W., Zhengzhi M., Hua Z. (2009). Aphrodisiac properties of *Allium tuberosum* seeds extract. *Journal of Ethnopharmacology*.

[11] Sharquie K. E., Al-Obaidi H. K. (2002). Onion juice (*Allium cepa* L.), a new topical treatment for alopecia areata. *The Journal of Dermatology*.

[12] Kim M. H., Choi Y. Y., Cho I. H., Hong J., Kim S. H., Yang W. M. (2014). *Angelica sinensis* induces hair regrowth via the inhibition of apoptosis signaling. *The American Journal of Chinese Medicine*.

[13] Choi E.-Y., Cho Y.-O. (2006). Allium vegetable diet can reduce the exercise-induced oxidative stress but does not alter plasma cholesterol profile in rats. *Annals of Nutrition and Metabolism*.

[14] Zhou Y., Zhuang W., Hu W., Liu G. J., Wu T. X., Wu X. T. (2011). Consumption of large amounts of *Allium* vegetables reduces risk for gastric cancer in a meta-analysis. *Gastroenterology*.

[15] Kang M., Kim H. J., Jayasena D. D. (2012). Effects of combined treatments of electron-beam irradiation and addition of leek (*Allium tuberosum*) extract on reduction of pathogens in pork jerky. *Foodborne Pathogens and Disease*.

[16] Zhang H., Mallik A., Zeng R. S. (2013). Control of Panama disease of banana by rotating and intercropping with Chinese chive (*Allium tuberosum* Rottler): role of plant volatiles. *Journal of Chemical Ecology*.

[17] Kim T., Park K., Jung H. S., Kong W.-S., Jeon D., Lee S. H. (2014). Evaluation of anti-atopic dermatitis activity of hypsizigus marmoreus extract. *Phytotherapy Research*.

[18] Choi J. S., Jeon M. H., Moon W. S. (2014). In vivo hair growth-promoting effect of rice bran extract prepared by supercritical carbon dioxide fluid. *Biological and Pharmaceutical Bulletin*.

[19] Shin D. H., Cha Y. J., Yang K. E. (2014). Ginsenoside rg3 up-regulates the expression of vascular endothelial growth factor in human dermal papilla cells and mouse hair follicles. *Phytotherapy Research*.

[20] Petiot A., Conti F. J. A., Grose R., Revest J. M., Hodivala-Dilke K. M., Dickson C. (2003). A crucial role for Fgfr2-IIIb signalling in epidermal development and hair follicle patterning. *Development*.

[21] Werner S., Smola H., Liao X. (1994). The function of KGF in morphogenesis of epithelium and reepithelialization of wounds. *Science*.

[22] Danilenko D. M., Ring B. D., Yanagihara D. (1995). Keratinocyte growth factor is an important endogenous mediator of hair follicle growth, development, and differentiation: normalization of the nu/nu follicular differentiation defect amelioration of chemotherapy-induced alopecia. *American Journal of Pathology*.

[23] Li J., Yang Z., Li Z., Gu L., Wang Y., Sung C. (2014). Exogenous IGF-1 promotes hair growth by stimulating cell proliferation and down regulating TGF-*β*1 in C57BL/6 mice *in vivo*. *Growth Hormone & IGF Research*.

[24] Weger N., Schlake T. (2005). IGF-I signalling controls the hair growth cycle and the differentiation of hair shafts. *Journal of Investigative Dermatology*.

[25] Hayashi Y., Yamamoto N., Nakagawa T., Ito J. (2013). Insulin-like growth factor 1 inhibits hair cell apoptosis and promotes the cell cycle of supporting cells by activating different downstream cascades after pharmacological hair cell injury in neonatal mice. *Molecular and Cellular Neuroscience*.

